# Thermal eye injuries from dermatologic laser treatments—an experimental study

**DOI:** 10.1007/s10103-023-03769-3

**Published:** 2023-04-22

**Authors:** Lynhda Nguyen, Nikolaus Seeber, Stefan W. Schneider, Katharina Herberger

**Affiliations:** 1 Laser Department, Department of Dermatology and Venereology, University Medical Center Hamburg-Eppendorf, Martinistrasse 52, 20246 Hamburg, Germany; 2 Joint Practice for Dermatology Dres. Peter/ Seeber/ Altheide, Wandsbeker Marktstrasse 48–50, 22041 Hamburg, Germany; 3 Department of Dermatology and Venereology, University Medical Center Hamburg-Eppendorf, Martinistrasse 52, 20246 Hamburg, Germany

**Keywords:** Laser safety, Thermal eye injuries, Metal eye shields, Ocular complications

## Abstract

In recent years, severe ocular complications after dermatological laser therapies have been reported. One hypothesis is thermal damage due to heating of the metal eye shields. The aim of the present study is to evaluate the safety of ocular metal eye shields during laser therapy of the periocular region. For the experimental study, porcine eyelids were exposed to continuously increasing laser energy and multiple pulses using a number of dermatologic laser systems. Temperature differences of the convex and concave surface of metal eye shields were constantly measured using a thermocouple. Maximum increase of the convex surface of shields was + 8.9 °C (± 0.1 °C) provided by the long-pulsed alexandrite laser (20–25-J/cm^2^ energy, 15-mm spot size, 20-ms pulse duration, 1 Hz). Present data indicate that metal eye shields provide sufficient thermal protection when clinically used laser parameters are applied. Other safety precautions continue to be essential to protect both the patient and the laser operator. These include the use of nonreflective metal eye shields, precise knowledge of laser physics, and a clear understanding of how they interact with ocular and periocular anatomy.

## Introduction

Laser procedures of the eyelid and periocular region are used by physicians of several specialties to treat various aesthetic and medical challenges. Over time, a large number of periorbital lesions have accumulated for approaches for laser treatment. The increasing use of periocular laser treatment has also been associated with a growing number of reports of severe ocular complications including cornea ulceration and retinopathy [1].

The most common procedures that led to laser-induced ocular injuries are skin resurfacing and epilation, especially of the eyebrows, the face, and the underarms. Furthermore, vascular and pigmented lesions such as port-wine stains, hemangiomas, and lentigines were reported in those cases. The most often reported laser systems causing eye injuries were carbon dioxide (CO_2_) lasers, followed by alexandrite lasers and neodymium-doped yttrium aluminum garnet (Nd:YAG) lasers [2].

An analysis on reported laser accidents found out that the eye accounted to the majority of involved injured organs [3]. Ocular injury from laser treatment can result in loss of vision or other serious damage [4]. For that reason, sufficient ocular safety measures are essential. For laser treatments of the periocular region, metal eye shields are recommended [5]. Photodisruptive (photomechanical) and photocoagulative (photothermal) effects are the two main mechanisms of damage from dermatologic laser treatments near the eye (Table 1). Few cases also reported on physical–mechanical irritation by the eye shield itself and chemical irritation by antiseptic solutions [6, 7]. Thermal conduction by the metal eye shields and subsequent thermal injuries have been discussed especially in current literature [1]. Wavelength, pulse duration, fluence, spot size, and frequency are parameters that determine the penetration depth in skin as well as the scope and lateral extent of temperature rise in exposed tissue [8, 9].

**Table 1:** Overview of mechanisms of eye injuries during laser treatments and safety measures

Mechanism of damage	Safety measures
Photocoagulative (photothermal) effect	• Sufficient eye protection, metal eye shields • Continuous contact/air cooling during laser treatment • Cautious choice of parameters, low laser frequency
Photodisruptive (photomechanical) effect	• Sufficient eye protection, metal eye shields • Avoidance of misdirection of laser beam • Cautious choice of parameters • Cautious application of lasers with high penetration depth, e.g., Nd:YAG lasers
Physical/mechanical irritation through eye shield itself	• Keeping time of wearing eye shields as short as possible • Usage of ophthalmological ointment
Dehydration of cornea	• Keeping time of wearing eye shields as short as possible • Usage of ophthalmological ointment
Antiseptic and irritative agent	• Thorough removal of antiseptic solution from eye shields

To date, there have been few studies that have evaluated the ability of those shields to protect against thermal ocular injuries. The purpose of this study is to investigate the safety of metal eye shields for laser treatments around the eye by measuring their temperature rise and transmission. To achieve this objective, we applied commonly used laser systems on porcine tissue.

## Materials and methods

### Design

**Fig. 1:**
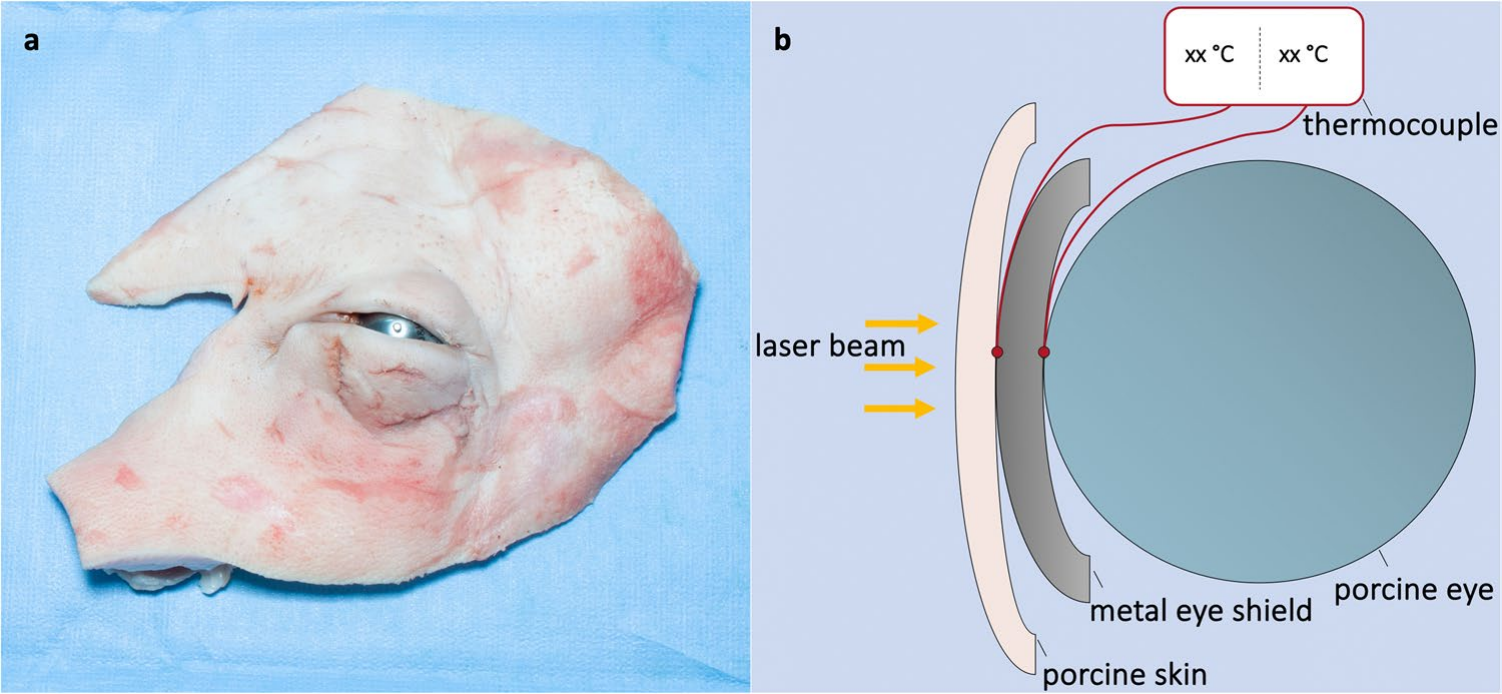
Experimental setup. **a** Porcine eye and surrounding tissue with inserted metal eye shield. **b** Schematic overview of experimental setup

This study used adult porcine cadaver eyes and eyelids that were obtained from the local abattoir and used within 5 h after enucleation (Fig. 1). For every laser system, eight tissue units were used. All eyes were stored at 4 °C and were used after they reached room temperature. To adjust to natural human conditions, eyelid skin was prepared and surgically thinned out by reducing the dermal tissue to an overall skin thickness of 1 mm [10]. A metal eye shield (Eye Shield 6–667, Duckworth & Kent Ltd.) was placed between the eye and the eye lid. To mimic natural conditions, an artificial tear film (Vidisic®, Bausch + Lomb GmbH) was applied on the shield. During laser irradiation, temperature difference Δ*T* of the convex and concave surface of the eye shield was continuously measured using a thermocouple with type K probes (UT320D, UNI-T®) with a temperature range of − 50 to + 1300 °C and a precision of 0.1 °C. As stated by the manufacturer, response time of thermocouples was four times per second. Laser irradiation took place on the convex side of the eye shield directly above the thermocouples.

### Laser irradiation procedure

The following laser systems were used: a pulsed dye and Q-switched Nd:YAG laser (Cynergy™, Cynosure®), a Q-switched ruby laser (Sinon II™, Alma Lasers®), a Q-switched Alexandrite laser (Elite MPX, Cynosure®), an Alexandrite picosecond laser (PicoSure Laser®, Cynosure®), an Nd:YAG picosecond laser (PicoSure Laser®, Cynosure®), an erbium-doped yttrium aluminum garnet (Er:YAG) laser (MCL29 Dermablate®, Aesculap Meditec®), and a CO_2_ laser (eCO_2_ Plus™, Lutronic®). Applied laser systems and parameters are demonstrated in Table 2. Energies corresponding to the usual treatment parameters were used. Radiant exposure was continuously increased and multiple pulses were applied to simulate maximal thermal effects. Subsequent cycles were applied after temperature decreased to room temperature. No cooling methods were applied.

**Table 2:** Overview of applied laser systems and parameters

Laser system	Energy [J/cm^2^]	Pulse duration [ms]	Spot size [mm]	Frequency [Hz]	Number of pulses [*n*]
595-nm pulsed dye laser (Cynergy™, Cynosure®)	8–20	0.5	5	2	5
694-nm ruby laser (Sinon II™, Alma Lasers®)	4–5	6	5	1	5
Long-pulsed 795 nm alexandrite laser (Elite MPX, Cynosure®)	20–25	20	15	1	5
Long-pulsed 1064-nm Nd:YAG laser (Cynergy™, Cynosure®)	30–120	20	5	1	5
Picosecond 795-nm alexandrite laser (PicoSure Laser®, Cynosure®)	2–10	0.55	5	1	5
Picosecond 1064-nm Nd:YAG laser (PicoSure Laser®, Cynosure®)	2–10	0.55	5	1	5
2940-nm Er:YAG laser (MCL29 Dermablate®, Aesculap Meditec®)	400–800	200,000	3	1	5
10,6000-nm CO_2_ laser (eCO_2_ Plus™, Lutronic®)	40–200	120 µm handpiece, 8 mm square, 15% density, 30 W			

### Statistical analysis

Statistical analysis was performed using Microsoft® Excel (Version 16.56, Microsoft Cooperation) and MATLAB® software (Version 9.11, The Mathworks Inc.). All data are represented as mean ± standard error of the mean.

## Results

**Fig. 2:**
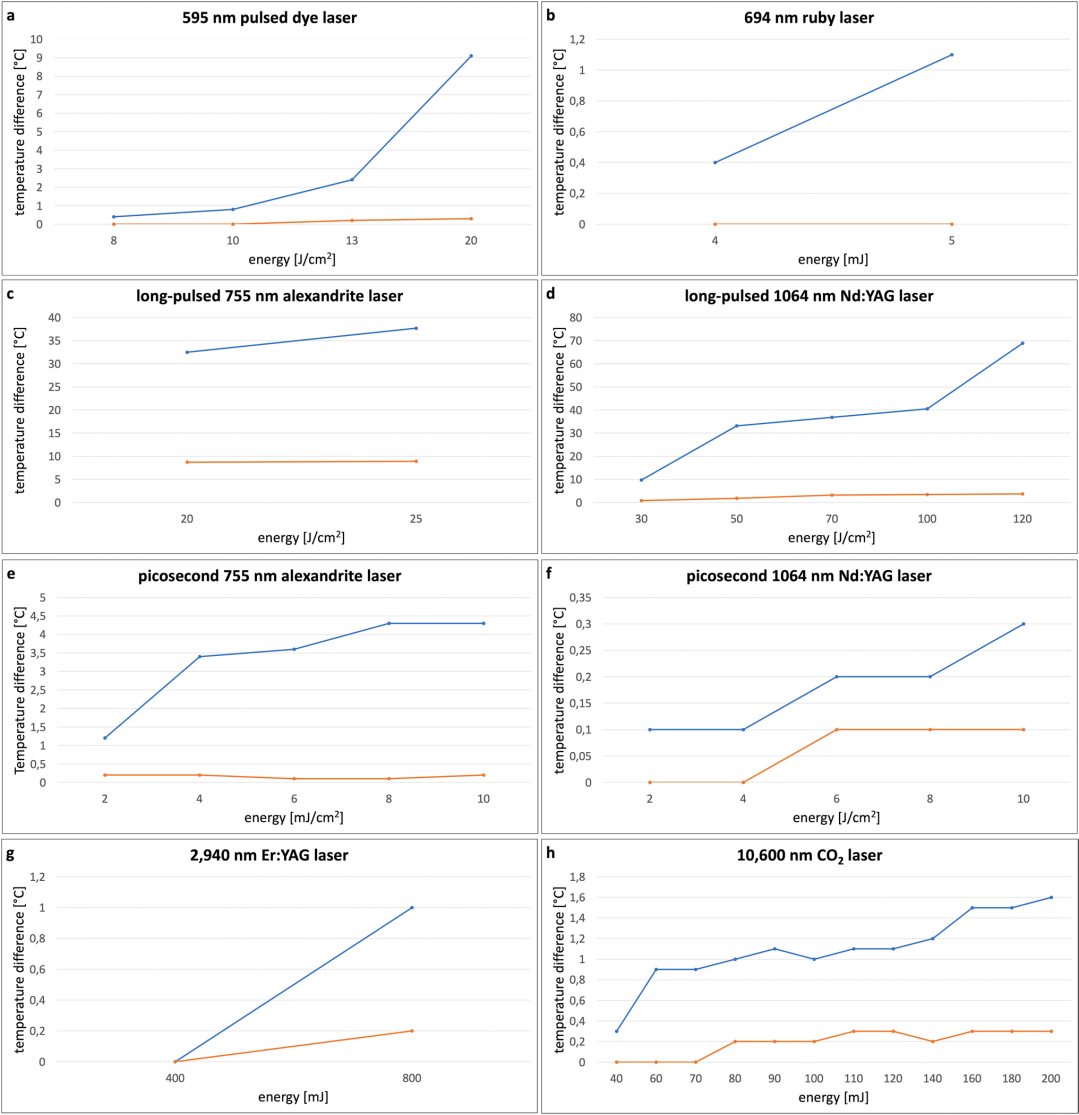
Temperature difference ΔT [°C] on energy [J/cm^2^] of the convex (blue) and concave (orange) surface of metal eye shields during laser irradiation with different laser systems. **a** Pulsed dye laser. **b** Ruby laser. **c** Long-pulsed alexandrite laser. **d** Long-pulsed Nd:YAG laser. **e** Picosecond alexandrite laser. **f** Picosecond Nd:YAG laser. **g** Er:YAG laser. **h** CO_2_ laser

Thermal response curves of the convex and concave surface of eye shields during laser irradiations with different laser systems are demonstrated in Fig. 2.

During treatment with the pulsed dye laser, a maximum Δ*T* of + 9.1 °C (± 0.2 °C) was measured on the convex surface and + 0.3 °C (± 0.1 °C) on the concave side. Ruby laser beams caused a Δ*T* of a maximum value of + 1.1 °C (± 0.1 °C) on the front surface. No significant temperature rise could be measured on the concave surface. The highest temperature rise on the concave surface was provided by applying the long-pulsed alexandrite laser. At an energy of 25 J/cm^2^, spot size of 15 mm, pulse duration of 20 ms and frequency of 1 Hz, a maximum ΔT of + 37.7 °C (± 0.3 °C) on the front side, and + 8.9 °C (± 0.1 °C) on the back side were observed. When using long-pulsed Nd:YAG laser, a maximum increase of + 68.9 °C (± 0.3 °C) was shown on the convex side and + 3.7 °C (± 0.1 °C) on the concave side. By applying the picosecond alexandrite laser, Δ*T* reached a maximum value of + 4.3 °C (± 0.1 °C) at an irradiation energy of 8 J/cm^2^ on the convex surface. Maximum temperature elevation on the back side was + 0.2 °C (± 0.1 °C) at an energy of 4 J/cm^2^. Picosecond Nd:YAG laser irradiation caused a maximum Δ*T* of + 0.3 °C (± 0.1 °C) on the convex surface and + 0.1 °C (± 0.1 °C) on the concave surface of the eye shield. Laser irradiation using the Er:YAG laser resulted in a maximum increase in temperature of 1.1 °C (± 0.2 °C) on the convex surface. On the concave side, a maximum Δ*T* of 0.2 °C (± 0.1 °C) was measured. During CO_2_ laser treatment, the front side showed a maximum temperature difference of + 1.0 °C (± 0.2 °C) and the back side a Δ*T* of + 0.1 °C (± 0.1 °C).

Overall, the temperature elevation at the concave surface of the eye shield was no more than 8.9 °C in any measurements.

## Discussion

In the present experimental study, temperature elevation of metal eye shields during laser treatments was investigated using various commonly used dermatological laser systems. We provided evidence that metal eye shields allow efficient protection of thermal injuries when applying periorbital laser treatments at clinical used parameters.

Laser photons are being absorbed by the tissue and converted to thermal energy. Depending on the temperature, various degrees of damage may occur. At 42 °C, conformation alteration of proteins can be observed. Protein denaturation occur at a temperature of about 60 °C [11]. A study by Peppers et al. (1969) indicated that thermal damage of the cornea starts at 67 °C [12]. Temperature differences of metal eye shields did not meet thermal value for protein denaturation despite application of high fluence and multiple laser pulses and omission of cooling methods. The maximum temperature elevation on the concave surface was 8.9 °C using the long-pulsed alexandrite laser (25-J/cm^2^ energy, 15-mm spot size, 20-ms pulse duration, 1-Hz frequency). We measured a marked temperature difference on the concave side of the eye shields of the long-pulsed alexandrite laser (8.9 °C) compared to the long-pulsed Nd:YAG laser (3.7 °C) with otherwise identical laser parameters. This observation may be due to the fact that epidermal pigmentation has a higher optical absorption coefficient at a wavelength of 755 nm than of 1064 nm [13–15]. We hypothesize that due to high reflection capacity of metal surface, laser pulses at clinically applied settings do not cause massive temperature changes on the concave side. However, this reflectivity requires sufficient protection for patients, the physician, and treatment team.

Eye comfort should be continuously monitored during treatment. As eye shields do not cover the whole frontal eye surface and eyelids often do not cover the whole eye shield area, misdirection of the laser beam should be avoided [4]. Treatment for laser-induced ocular injuries is very limited making prevention essential. Plastic eye shields, especially dark-colored ones, are not recommended as they may not withstand thermal and mechanical impact of pulsed lasers [16, 17].

Due to anatomical factors, the human eye is at risk for severe injuries during laser therapy of the periocular region. The thin eyelid may act favorable for thermal transfer through metal eye shields and corneal epithelium. Reviews by Huang et al. (2018) and by Juhasz et al. (2021) revealed that in about 60% of reported cases, no proper eye protection or no further information on eye protection was provided [1, 2]. This indicates growing evidence that insufficient safety measures are the cause for severe injuries in most cases. The British Medical Laser Association reported that 67% of laser injuries were caused by operator error [3]. This highlights the importance of knowledge about and provision of safety measures.

However, photothermal damage is not the only photophysical process of eye injuries. Lasers with high penetration depth like 1064-nm Nd:YAG lasers may lead to absorption by pigment-rich structures of the eye, e.g., the iris and retinal epithelial membrane, and cause photodisruptive (photomechanical) effects through generation of explosive shock [1, 18]. Several cases reported on the usage of chlorhexidine as an antiseptic agent for eye shields in some practices. This malpractice may result in corneal defects of various degrees ranging from corneal edema, stromal scarring to endothelial destruction when not removed properly before insertion under the eyelid [6, 7, 19, 20]. In one report, a patient experienced corneal irritation by the eye shield itself [21]. The latter can occur through increased eye movement during laser treatment and rubbing of the cornea against the shell. Wearing of metal shields for too long can also cause the corneal epithelium to dry out. Therefore, period of wearing should be kept as short as possible and moisturization should be ensured, e.g., by using ophthalmological ointment.

To enable rapid ophthalmological interventions and thus reduce associated morbidity of patients, laser physicians should be aware of symptoms and signs associated with eye injuries. Symptoms occur immediately or shortly after laser treatment and present as visual abnormalities and pain followed by photophobia and visual glare [2]. Most common ophthalmologic findings were hyperemia of the eye and pupil irregularities [22]. Other reported ophthalmic injuries include corneal abrasion and ulceration, and photocoagulation to the lens capsule and retina [23, 24]. Temporary, lower eyelid ectropion was the most commonly reported complication associated with periocular CO_2_ laser treatments [25, 26].

The findings of this study have to be seen in light of some limitations. First, post-mortem tissue was used which lack in blood circulation. Thus, low quantity of hemoglobin targeted by pulsed dye lasers may restrict interpretation of data. Second, porcine tissue was used at room temperature. Nevertheless, for the objective, temperature difference was the critical measure which could be provided in the experimental setup. Furthermore, due to the nature of experiments on animal tissue, interspecies differences may lead to limited generalization of the results.

The available data underline the importance of metal eye shields for periorbital laser treatments. In conclusion, this study indicates sufficient thermal protection by metal eye shields during laser treatment at clinically used parameters. Despite precautions, ocular laser injury might occur, though rarely. Laser therapy in the periorbital region requires a high level of expertise and caution and should only be performed by qualified and trained physicians, e.g., board-certified dermatologists, plastic and aesthetic surgeons, and ophthalmologists. Further investigations are warranted to analyze ocular injuries by photodisruptive mechanisms and to clarify in detail prevalence and systematic data on laser-based eye injuries.

## Data Availability

The authors confirm that the data supporting the findings of this study are available in the article.
